# Supplementary material for: Persistence, period and precision of autonomous cellular oscillators from the zebrafish segmentation clock
Source: eLife. 2016 Feb 13;5:e08438. doi: 10.7554/eLife.08438 (PMC4803185; doi:10.7554/eLife.08438)
Supplement: Figure 1—source data 1. — Summary of statistics of time series traces recorded and analyzed in vitro in tailbud explants or tailbud cells. Peaks were identified, and the period/amplitude of cycles was determined as described in Materials and methods. A maximum period is defined in the method at 140 min, approximately twice the mean. Serum only cells were from the same cell suspension as those that were then treated with Fgf8b for the experiments 280711 and 250112. Pooled data from N = 2 independent cultures, for a total of n = 52 serum only cells. Pooled data from N = 4 independent cultures, for a total of n = 149 Fgf8b treated cells. To culture fully isolated cells, a cell suspension was serially diluted in wells within a 96-well plate, producing wells with a single tailbud cell. These were also treated with Fgf8b. N = 5 independent 96-well plate experiments, with a total of n = 10 fully isolated cells in these experiments. DOI: http://dx.doi.org/10.7554/eLife.08438.004 [file elife-08438-fig1-data1.docx]

|  | **peaks per explant** | **period [min]** | **amplitude [a.u.]** | **peaks per cell** | **period [min]** | **amplitude [a.u.]** | **peaks per cell** | **period [min]** | **amplitude [a.u.]** | **peaks per cell** | **period [min]** | **amplitude [a.u.]** |
| --- | --- | --- | --- | --- | --- | --- | --- | --- | --- | --- | --- | --- |
|  | ***Explant*** | | | ***Serum only*** | | | ***Serum + Fgf8b***  ***Low density*** | | | ***Serum + Fgf8b***  ***Fully isolated*** | | |
| **min / max** | 7 / 10 | 26 / 70 | 17.1 / 614.8 | 1 / 6 | 62 / 118 | 34.3 / 751.6 | 1 / 8 | 46 / 140 | 16.6 / 923.9 | 2 / 7 | 38 / 124 | 3 / 362.8 |
| **median** | 8 | 40 | 88 | 2 | 78 | 200.8 | 5 | 76 | 127.5 | 6 | 56 | 33.3 |
| **mean** | 8.3 | 42.5 | 155.9 | 2.6 | 81.8 | 223.2 | 4.7 | 78.7 | 155.7 | 5.3 | 62.1 | 70.6 |
| **variance** | 2.3 | 130.4 | 23128 | 1.6 | 152.6 | 17825 | 2.5 | 231.8 | 14647 | 2.9 | 460.5 | 6632.4 |
| **std** | 1.5 | 11.4 | 152.1 | 1.3 | 12.4 | 133.5 | 1.6 | 15.2 | 121.0 | 1.7 | 21.5 | 81.4 |
| **sem** | 0.9 | 2.4 | 32.4 | 0.2 | 1.5 | 15.8 | 0.1 | 0.7 | 5.6 | 0.5 | 3.4 | 13 |
| **95% CI** | 5.8 - 10.9 | 38.4 - 46.7 | 100.1 - 211.7 | 2.3 - 2.9 | 79.4 - 84.2 | 196.8 - 249.6 | 4.4 - 4.9 | 77.5 - 79.9 | 146.5 - 164.8 | 4.3 – 6.3 | 56.3 – 67.9 | 48.6 – 92.6 |

**Webb et al., Figure 1-source data 1**

**Figure 1-source data 1. Summary table of segmentation clock tissue and cellular oscillatory properties.** Summary of statistics of time series traces recorded and analyzed *in vitro* in tailbud explants or tailbud cells. Peaks were identified, and the period / amplitude of cycles was determined as described in **Materials and methods**. A maximum period is defined in the method at 140 min, approximately twice the mean. Serum only cells were from the same cell suspension as those that were then treated with Fgf8b for the experiments 280711 and 250112. Pooled data from *N* = 2 independent cultures, for a total of *n* = 52 serum only cells. Pooled data from *N* = 4 independent cultures, for a total of *n* = 149 Fgf8b treated cells. To culture fully isolated cells, a cell suspension was serially diluted in wells within a 96-well plate, producing wells with a single tailbud cell. These were also treated with Fgf8b. *N = 5* independent 96-well plate experiments, with a total of *n* = 10 fully isolated cells in these experiments.
