# Supplementary material for: Persistence, period and precision of autonomous cellular oscillators from the zebrafish segmentation clock
Source: eLife. 2016 Feb 13;5:e08438. doi: 10.7554/eLife.08438 (PMC4803185; doi:10.7554/eLife.08438)
Supplement: Figure 1—source data 2. — Description of in vitro cultured tailbud cell population treated with Fgf8b (n = 547), using multiple donor embryos in each of 4 independent experimental replicates (N = 4), carried out on separate days. Across the 29 fields recorded, we observed cell divisions in both YFP-negative (30, 5% of total cells) and YFP-positive cells (13, 2% of total cells). We found a range in the number of cell divisions from 0 to 5 cells per field, with an average of 1.5 (±1 SD) divisions per field. The categories of disqualification list the first event in a recording that led to disqualification. For example, four divisions in YFP-positive cells occurred after the cell had been disqualified for another reason (movement in and out of field, touching another cell). DOI: http://dx.doi.org/10.7554/eLife.08438.005 [file elife-08438-fig1-data2.docx]

**Webb et al., Figure 1-source data 2**

| ***Experiments, N=4*** | *280711* | *250112* | *070312* | *251012* |
| --- | --- | --- | --- | --- |
| **Fields of view** | 10 | 7 | 6 | 6 |
| **Total cells,** *n=547* | 190 | 131 | 101 | 125 |
| **Total YFP –ve cells** | 104 | 47 | 27 | 48 |
| **Total YFP +ve cells** | 86 | 84 | 74 | 77 |
| **Total disqualified cells:** | 42 | 24 | 29 | 40 |
| **Moved** | 7 | 6 | 3 | 4 |
| **Touched other cell** | 28 | 6 | 11 | 25 |
| **Divided** | 2 | 1 | 4 | 2 |
| **Died** | 5 | 11 | 11 | 9 |
| **YFP+ does not oscillate** | 3 | 14 | 15 | 7 |
| **YFP+ oscillates** | 41 | 46 | 30 | 30 |

**Figure 1-source data 2. Summary table of low-density segmentation clock cell experiments.** Description of *in vitro* cultured tailbud cell population treated with Fgf8b (*n*=547), using multiple donor embryos in each of 4 independent experimental replicates (*N*=4), carried out on separate days. Across the 29 fields recorded, we observed cell divisions in both YFP-negative (30, 5% of total cells) and YFP-positive cells (13, 2% of total cells). We found a range in the number of cell divisions from 0 to 5 cells per field, with an average of 1.5 (±1 SD) divisions per field. The categories of disqualification list the *first* event in a recording that led to disqualification. For example, four divisions in YFP-positive cells occurred after the cell had been disqualified for another reason (movement in and out of field, touching another cell).
