# Supplementary figures and images for: Persistence, period and precision of autonomous cellular oscillators from the zebrafish segmentation clock
Source: eLife. 2016 Feb 13;5:e08438. doi: 10.7554/eLife.08438 (PMC4803185; doi:10.7554/eLife.08438)

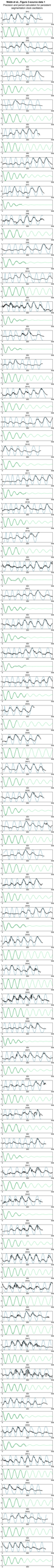

Supplement: Figure 3—source data 1. — Each set of panels shows, successively, the background-subtracted average YFP intensity levels over time from a single persistently oscillating cell in black; the cosine of the phase calculated from the wavelet transformation in blue; and the autocorrelation function in green. The dashed green curve shows the analytical fit of the autocorrelation. Both period and quality factor can be calculated from this procedure (see Supplementary file 1). This is the complete persistent cell data set, a sub-set of the low-density set, from which the plots of period andquality factor QP in Figure 3B and D are generated. DOI: http://dx.doi.org/10.7554/eLife.08438.023 [file elife-08438-fig3-data1.pdf]

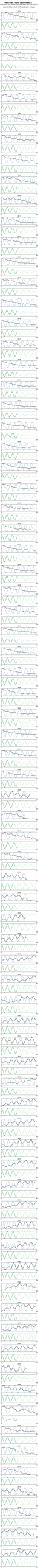

Supplement: Figure 3—source data 2. — As for data set supplement 3–1, each set of panels shows, successively, the background-subtracted average YFP intensity levels from a region of posterior PSM tissue in a Looping embryo in black; the cosine of the phase calculated from the wavelet transformation in blue; and the autocorrelation function in green. The dashed green curve shows the analytical fit of the autocorrelation. Both period and quality factor can be calculated from this procedure. The original intensity versus time data comes from Soroldoni et al. (2014). This is the complete dataset from time-lapse data of 24 embryos from which the plot of quality factor QEmbryo in Figure 3B is generated. DOI: http://dx.doi.org/10.7554/eLife.08438.024 [file elife-08438-fig3-data2.pdf]

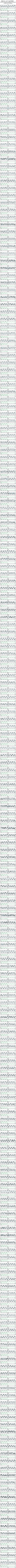

Supplement: Figure 3—source data 3. — As for data set supplement 3–1, each set of panels shows, successively, the background-subtracted intensity levels from a single persistently oscillating Per2-Lucifcerase-expressing fibroblast over time in black; the cosine of the phase calculated from the wavelet transformation in blue; and the autocorrelation function in green. The dashed green curve shows the analytical fit of the autocorrelation. Both period and quality factor can be calculated from this procedure. The original intensity versus time data comes from Leise et al. (2012). This is the complete fibroblast dataset from which the plot of quality factor QF in Figure 3D is generated. DOI: http://dx.doi.org/10.7554/eLife.08438.025 [file elife-08438-fig3-data3.pdf]
