# Supplementary material for: Persistence, period and precision of autonomous cellular oscillators from the zebrafish segmentation clock
Source: eLife. 2016 Feb 13;5:e08438. doi: 10.7554/eLife.08438 (PMC4803185; doi:10.7554/eLife.08438)
Supplement: Supplementary file 1. — DOI: http://dx.doi.org/10.7554/eLife.08438.027 [file elife-08438-supp1.pdf]

Supplementary Materials for

# Persistence, period and precision of autonomous cellular oscillators from the zebrafish segmentation clock

**Alexis B. Webb[1,2,3], Iván Lengyel[4], David J. Jörg[5], Frank Jülicher[5], Luis G. Morelli[4], Andrew C. Oates[1,2,3,6,*]**

[1]MRC National Institute for Medical Research, The Ridgeway, Mill Hill, London, NW7 1AA, UK
[2]Current Address: Francis Crick institute, Mill Hill Laboratory, The Ridgeway, NW7 1AA London, UK
[3]Max Planck Institute of Molecular Cell Biology and Genetics, Pfotenhauerstr. 108, 01307 Dresden, Germany
[4]Departamento de Física, FCEyN UBA and IFIBA, CONICET; Pabellón 1, Ciudad Universitaria, 1428 Buenos Aires, Argentina
[5]Max Planck Institute for the Physics of Complex Systems, Nöthnitzer Str. 38, 01187 Dresden, Germany
[6]Department of Cell and Developmental Biology, University College London, Gower Street, London, WC1E 6BT, UK

[*]Correspondence to Andrew.Oates@crick.ac.uk

# Contents

**Supplementary Text** **2**
**Stuart-Landau theory with slow fluctuations** **2**
**Quantification of noisy oscillations** **3**
Fraction of time that cells oscillate 3
Quality factor through wavelet transforms 3
**Numerical methods** **6**
Solution of Stuart-Landau equation with color noise in $\mu$ and additive white noise 7

# Supplementary Text

## Stuart-Landau theory with slow fluctuations

The data shows oscillations with slow amplitude changes during the course of the timeseries, Main Text Figure 2. In some cases the amplitude of oscillations seems to fade out, in others oscillations seem to start at an intermediate point in the time series. These slow amplitude changes in the data are accompanied by period fluctuations as described by the quality factor, Main Text Figure 3. Here we propose a generic theory that captures these features. The theory is based on Stuart-Landau oscillators, and we introduce additive noise and slow fluctuations in parameter values to describe the features observed in the data. The Stuart-Landau (SL) equation is the normal form near a Hopf bifurcation (Pikovsky et al., 2001). We choose this particular normal form because close to the supercritical Hopf bifurcation amplitude is sensitive to changes in parameters, which could explain the observed amplitude fluctuations. Furthermore, in several biochemical descriptions of the segmentation clock, the center manifold reduction leads to the normal form of a supercritical Hopf bifurcation (Verdugo & Rand, 2008b,a; Wu & Eshete, 2011). Thus, we describe the onset of noisy oscillations through a Hopf bifurcation for a complex variable $z$ as

$$\frac{\mathrm{d}z}{\mathrm{d}t} = (\mu(t) + i\omega)z - (b + iq)z|z|^2 + \xi_z(t) \quad , \tag{S1}$$

with a time dependent real parameter

$$\mu(t) = \langle \mu \rangle + \xi_\mu(t) \quad , \tag{S2}$$

constant real parameters $\omega$, $b$, $q$, and additive noise $\xi_z(t) = \xi_x(t) + i\xi_y(t)$. The noise $\xi_\mu(t)$ is a color noise term with zero mean, variance $\sigma_\mu^2$ and autocorrelation time $\tau_\mu$,

$$\begin{aligned} \langle \xi_\mu(t) \rangle &= 0 \\ \langle \xi_\mu(t)^2 \rangle &= \sigma_\mu^2 \\ \langle \xi_\mu(t)\xi_\mu(t') \rangle &= \sigma_\mu^2 e^{-|t-t'|/\tau_\mu} \quad . \end{aligned} \tag{S3}$$

Thus, $\mu(t)$ has mean $\langle \mu \rangle$ and fluctuates with variance $\sigma_\mu^2$ and characteristic time $\tau_\mu$. The additive noise $\xi_z(t)$ is an uncorrelated white noise, with uncorrelated and symmetric real and imaginary components

$$\begin{aligned} \langle \xi_x(t) \rangle = \langle \xi_y(t) \rangle &= 0 \\ \langle \xi_x(t)^2 \rangle = \langle \xi_y(t)^2 \rangle &= \sigma_z^2 \\ \langle \xi_x(t)\xi_x(t') \rangle = \langle \xi_y(t)\xi_y(t') \rangle &= \sigma_z^2 \delta(t - t') \,, \end{aligned} \tag{S4}$$

and

$$\langle \xi_x(t)\xi_y(t) \rangle = 0 \,. \tag{S5}$$

**Polar form of the SL equation.** The complex variable $z$ can be cast in its polar form $z = re^{i\theta}$, where $r \equiv r(t)$ is an amplitude and $\theta \equiv \theta(t)$ a phase. The polar form of the SL Eq. (S1) is

$$\frac{\mathrm{d}r}{\mathrm{d}t} = \mu r \left(1 - \frac{b}{\mu} r^2\right) , \tag{S6}$$

$$\frac{\mathrm{d}\theta}{\mathrm{d}t} = \omega - q r^2 . \tag{S7}$$

These two equations describe the time evolution of amplitude and phase respectively. Oscillations can occur in the system depending on the value of $\mu$. For $\mu < 0$ the amplitude decays to zero and the dynamics settles after some time to a fixed point at the origin. For $\mu > 0$ the system oscillates: the amplitude settles to the non-zero value $r_* = \sqrt{\mu/b}$ while the phase grows at a rate $\dot{\theta} = \omega - q\, r_*^2$, Main Text Figure 2D. The non-isochronicity $q$ describes amplitude effects on phase dynamics. For $q = 0$ the frequency of oscillations is $\omega$, independent of the amplitude. For $q > 0$ the autonomous frequency of oscillators depends on amplitude, and the flow is sheared away from the limit cycle (Montbrió & Pazó, 2011). Motivated by a lack of correlation between period and amplitude in the data, in this work we consider the isochronous case $q = 0$ where amplitude fluctuations do not affect the precision of oscillations.

## Quantification of noisy oscillations

As a consequence of fluctuations, noisy oscillations show amplitude variations and impaired precision. Below, we discuss the methods we use in the Main Text to make a quantitative assessment of these features observed in the data.

### Fraction of time that cells oscillate

Single cells in the low-density dataset show a remarkable variety of behaviors, including cells that start or stop oscillating during the experiment, Main Text Figure 2A and Figure supplement 1-5. To quantitate this aspect of variability, we introduce the fraction of time $F$ that cells spend oscillating, as measured by the number of peaks occurring over time given the observed mean period. For a mean period $\langle T \rangle$ and a fixed time interval $\Delta T$, we define the fraction of time $F$ that cells are oscillating as the ratio of the number of peaks $\nu$ actually observed during the fixed time $\Delta T$, to the average number of possible peaks in this interval $\Delta T / \langle T \rangle$:

$$F = \frac{\nu \, \langle T \rangle}{\Delta T} \quad . \tag{S8}$$

Values of $F$ close to 1 indicate that fluctuations in the number of cycles are low, and $F < 1$ indicates that cells that do not oscillate in some part of the trace. For example $F \approx 0.5$ indicates that the cell oscillates only half of the time in the interval $\Delta T$.

### Quality factor through wavelet transforms

The quality factor is a measure of the precision of an oscillator, that is, for the number of oscillation cycles over which period fluctuations are small. Usually, the quality factor is determined by

directly calculating the autocorrelation function of an oscillatory time series (Morelli & Jülicher, 2007). However, the short time series analyzed here show large amplitude variations which introduce a spurious weighting effect to the autocorrelation. This impedes the direct use of the autocorrelation for precision measurements. Instead, we first generate a phase time series of the oscillatory time series using a wavelet transform as explained below. This phase time series is independent of the oscillation amplitude. We subsequently compute the autocorrelation of the phase time series to obtain the quality factor.

**Wavelet transform.** The wavelet transform is a method to systematically obtain a phase signal $\phi$ from an oscillatory time series $X$ (Quian Quiroga et al., 2002). For a discrete time series $X = (X_1, \ldots, X_n)$ sampled with time interval $\varepsilon$, where $X_k$ corresponds to the time point $t = \varepsilon k$, the continuous wavelet transform is given by

$$W_\sigma(k) = \frac{1}{\sqrt{\sigma}} \sum_{j=1}^{n} X_j \, \Psi^*\left(\frac{j-k}{\sigma}\right) , \tag{S9}$$

where $\sigma$ is the wavelet scale. The inverse $\sigma^{-1}$ is a dimensionless frequency and plays a role comparable to the frequency in a Fourier transform. The so-called mother wavelet $\Psi(\tau)$ is a complex-valued oscillatory function that decays for large $|\tau|$ (Torrence & Compo, 1998). $\Psi^*$ denotes the complex conjugate of $\Psi$. Here we employ the Paul wavelet function of order 4 (Torrence & Compo, 1998),

$$\Psi(\tau) = \sqrt{\frac{128}{35\pi}} \frac{1}{(1-\mathrm{i}\tau)^5} . \tag{S10}$$

The Paul wavelet function provides a high time resolution and is therefore particularly suited to resolve the temporal features of oscillatory time series with substantial period fluctuations (Torrence & Compo, 1998). The wavelet transform $W_\sigma$ depends on $\sigma$ and $k$ and can be expressed in terms of its magnitude and phase,

$$W_\sigma(k) = R_\sigma(k) \, \mathrm{e}^{\mathrm{i}\phi_\sigma(k)} . \tag{S11}$$

We interpret the phase $\phi_\sigma(k)$ as the phase of oscillation at the time scale corresponding to the wavelet scale $\sigma$ (Quian Quiroga et al., 2002). Hence, we choose the wavelet scale $\sigma$ to be close to the characteristic oscillation period of the time series $X$.[1] The resulting phase time series $\phi_\sigma(k)$ is nevertheless robust under small variations of the wavelet scale $\sigma$. In non-periodic time series, the finite width of the wavelet introduces spurious edge effects in the vicinity of the the left and right boundaries of the time series. This region is called the 'cone of influence' or COI (Torrence & Compo, 1998). To avoid these unwanted effects, we exclude the COI from the used time range for the phases. For the Paul wavelet with wavelet scale $\sigma$, the width of the COI is given by $\sigma/\sqrt{2}$.

[1] Wavelet scales are parameterized in an equal-tempered scheme,

$$\sigma_{\mu\nu} = 2^{\mu-1+\nu/\bar{\nu}} T_{\mathrm{P}}^{-1} , \tag{S12}$$

where $\mu$ is called the octave number, $\nu$ the voice number, $\bar{\nu}$ is the number of voices per octave, and $T_{\mathrm{P}}$ is the so-called Fourier period of the Paul wavelet function, Eq. (S10), given by $T_{\mathrm{P}} = 4\pi/9$ (Torrence & Compo, 1998).

| Dataset | Voices $\bar{\nu}$ per octave | Set of wavelet scales $(\mu, \nu)$ used for averages |
|---|---|---|
| 3–1 | 10 | (5,4), (5,5), (5,6), (5,7), (5,8), (5,9), (5,10), (6,1), (6,2) |
| 3–2 | 10 | (5,7), (5,8), (5,9), (5,10), (6,1), (6,2), (6,3), (6,4), (6,5) |
| 3–3 | 10 | (3,1), (3,2), (3,3), (3,4), (3,5), (3,6), (3,7), (3,8), (3,9) |

**Table S1** Wavelet scales parametrized by Eq. (S12) used to average over for the different supplementary datasets

**Oscillation period and quality factor.** From the phase signal $\phi$, we obain an oscillatory signal with constant amplitude,

$$\bar{X}_\sigma(k) = \cos\phi_\sigma(k) . \tag{S13}$$

To obtain the oscillation period and the quality factor of a time series, we compute the normalized autocorrelation function $A(k)$ of the oscillatory signal $\bar{X}_\sigma(k)$, Eq. (S13). For a given wavelet scale $\sigma$, it is given by

$$A(k) = \frac{\langle \bar{X}(k'+k)\bar{X}(k')\rangle - \langle \bar{X}\rangle^2}{\langle \bar{X}^2\rangle - \langle \bar{X}\rangle^2} , \tag{S14}$$

where $\langle\cdot\rangle$ denotes the average taken over the time index $k'$. The autocorrelation function typically has a functional form of the type

$$\mathcal{A}(t) \sim \mathrm{e}^{-t/t_\mathrm{c}} \cos\left(\frac{2\pi}{T}t\right) , \tag{S15}$$

an exponentially damped sinusoid, where $t_\mathrm{c}$ is the correlation time, $T$ is the period of oscillations, and $t$ is a continuous time variable, which is related to the discrete time index $k$ by $t = \varepsilon k$ with $\varepsilon$ being the time spacing of the original time series. We obtain $t_\mathrm{c}$ and $T$ by fitting the function (S15) to the experimentally obtained autocorrelation (S14). The quality factor $Q$ is defined as the dimensionless ratio of the correlation time and the period,

$$Q = \frac{t_\mathrm{c}}{T} . \tag{S16}$$

It measures the number of oscillations over which period fluctuations are small and thus serves as a measure for oscillator precision.

We apply this procedure for a set of wavelet scales $\sigma$ centered around the characteristic period of oscillations and take averages of the period $T$ and the quality factor $Q$ to ensure robustness of the results. The set of wavelet scales used for the respective supplementary datasets is provided in Table S1.

**Precision and quality factor of simulated timeseries.** To study the effect of additive noise on the precision of oscillations we do not include color noise in parameter $\mu$, as we are interested in how additive noise affects precision. In the analysis of experiments, we use wavelet transforms to

avoid amplitude variations and produce a phase time-series, from which we compute the quality factor. In the case of numerical simulations, we keep the amplitude constant so we can compute the phase of the oscillation explicitly. For this reason we do not need to perform a wavelet transform on the simulated traces before computing the autocorrelation function.

We base our analysis on time series that contain 6.5 complete cycles, similar to the average persistent cell in the persistent cells subset (see discussion below about quality factor estimation from short time series). Thus, we generate numerical simulations of 507 min $= 6.5 \times 78$ min, with the approach described below, see Eq. (S30). We vary $\sigma_z^2$ to generate fluctuations of different strengths, and compute the quality factor $Q$ with the same approach described for the data, Main Text Figure 3E.

**Accuracy of the quality factor estimate.** We use the quality factor $Q$ as a measure of the precision of oscillations, and compare the precision of single cells to other systems and to the theory, Main Text Figure 3. The value of the quality factor may depend on the length of the time series that is used to compute the autocorrelation function, Main Text Figure supplement 3-1. However, the qualitative comparison of the quality factor between two conditions $A$ and $B$ does not change for the range of parameters we study here: if $Q_A > Q_B$ for a given time series length, then $Q_A > Q_B$ consistently for any length, Main Text Figure supplement 3-1. This consistent behaviour allows us to compare different experiments and conditions, as long as we consider time windows which have the same number of cycles.

## Numerical methods

**Generating white noise.** We generate additive white noise with statistics (S4–S5) as

$$\xi(t) = \sigma_z w(t) \ , \tag{S17}$$

where $w$ is a Markovian, uncorrelated unit normal distribution, see (S19) below.

**Generating color noise.** We generate color noise with statistics (S3) by solving the Stochastic Differential Equation (SDE) of an Ornstein-Uhlenbeck process

$$\frac{\mathrm{d}\xi}{\mathrm{d}t} = -\frac{1}{\tau}\xi(t) + \sqrt{D}w(t) \quad , \tag{S18}$$

where $w$ is a Markovian, zero mean and uncorrelated Gaussian process

$$\begin{aligned} \langle w(t) \rangle &= 0 \quad , \\ \langle w(t) w(t') \rangle &= \delta(t - t') \quad . \end{aligned} \tag{S19}$$

The analytical solution of the Ornstein-Uhlenbeck process (S18) is (Gardiner, 2004)

$$\xi(t) = \xi(0)\mathrm{e}^{-t/\tau} + \sqrt{D} \int_0^t \mathrm{e}^{-(t-t')/\tau} w(t) \, \mathrm{d}t \, . \tag{S20}$$

If the initial condition is Gaussian distributed with mean $\langle\xi(0)\rangle$ and variance $\sigma^2(0)$, the $\xi(t)$ is Gaussian with mean, variance and time correlation

$$\langle\xi(t)\rangle = \langle\xi(0)\rangle \mathrm{e}^{-t/\tau} \tag{S21}$$

$$\mathrm{var}(\xi(t)) = \left(\sigma^2(0) - 2D\tau\right)\mathrm{e}^{-2t/\tau} + 2D\tau \tag{S22}$$

$$\langle\xi(t)\xi(s)\rangle = \left(\sigma^2(0) - 2D\tau\right)\mathrm{e}^{-(t+s)/\tau} + 2D\tau\mathrm{e}^{-|t+s|/\tau} \tag{S23}$$

Choosing

$$D = 2\sigma^2/\tau \tag{S24}$$

and the initial condition

$$\xi(0) = \sigma w(0)\ , \tag{S25}$$

the solution $\xi(t)$ has the properties (S3).

**Solution of Stuart-Landau equation with color noise in $\mu$ and additive white noise**

While the polar form is useful in some calculations, the phase becomes ill-defined when $r = 0$ and this may pose problems for numerical integration schemes. Therefore, in numerical simulations we use the cartesian form $z = x + iy$ where $x$ and $y$ are the real and the imaginary part of $z$. The polar and cartesian forms are connected by the transformation $x = r\cos\theta$ and $y = r\sin\theta$, and the cartesian form of the Stuart-Landau Eq. (S1) is

$$\frac{\mathrm{d}x}{\mathrm{d}t} = x\left(\mu - b(x^2+y^2)\right) + y\left(-\omega + q(x^2+y^2)\right)\ , \tag{S26}$$

$$\frac{\mathrm{d}y}{\mathrm{d}t} = x\left(\omega - q(x^2+y^2)\right) + y\left(\mu - b(x^2+y^2)\right)\ . \tag{S27}$$

Numerical integration is straightforward in this form since this system is well defined for all values of $x$ and $y$.

We generate numerical trajectories of the SDE with color noise (S18) with the following recurrence relation (Miguel & Toral, 1997)

$$\begin{aligned} \xi_\mu(0) =& \ \sigma_\mu w(0) \\ \xi_\mu(t+h) =& \ \xi_\mu(t)\mathrm{e}^{-h/\tau} + \sqrt{(1-\mathrm{e}^{-h/\tau})}\sigma_\mu w(t+h) \end{aligned} \tag{S28}$$

Noise generated by this process has properties (S21–S23).

White noise is generated using a pseudo random number generator for a unit normal distribution $w$

$$\begin{aligned} \xi_x =& \ \sigma_z w_x(t) \\ \xi_y =& \ \sigma_z w_y(t) \quad , \end{aligned} \tag{S29}$$

where $w_x$ and $w_y$ have the same statistics but are independent.

Redefining parameter $\mu$ as in (S2), and discretizing time $t_i = ih$ and variables $x(t_i) = x_i$ for $i = 0, \dots, n$, we generate numerical simulations of the Stuart-Landau Oscillator (S1) with color (S3) and additive white noise (S4-S5) with the following system of equations

$$\begin{aligned} x_{n+1} &= x_n + h\left[x_n\left(\mu_n - b(x_n^2 + y_n^2)\right) - y_n\omega\right] + \sigma_z w_{x_n} \\ y_{n+1} &= y_n + h\left[x_n\omega + y_n\left(\mu_n - b(x_n^2 + y_n^2)\right)\right] + \sigma_z w_{y_n} \\ \mu_n &= \mu + \xi_{\mu_n} \\ \xi_{\mu_{n+1}} &= \xi_{\mu_n} \mathrm{e}^{-h/\tau_\mu} + \sqrt{(1 - \mathrm{e}^{-h/\tau_\mu})}\sigma_\mu w_{n+1} \end{aligned} \tag{S30}$$

We set initial conditions

$$\begin{cases} x_0 = \mu \sin(u_{2\pi}) \\ y_0 = \mu \cos(u_{2\pi}) \\ \xi_{\mu_0} = \sigma_\mu w_0 \end{cases} \tag{S31}$$

where $u_{2\pi}$ is the uniform distribution in $[0, 2\pi)$.

The integration step $h$ has to be kept smaller than the correlation time $\tau$. This limitation arises from the way we include the noise in the equations using Euler's method. However, this approach lets us include color noise in $\mu$ and additive noise simultaneously. If one is interested in small correlation times it may be better to turn to Runge-Kutta methods, with the limitation is that only one noise can be included in the equations (Miguel & Toral, 1997).

# References


Gardiner, C. (2004). *Handbook of stochastic methods* (Third ed.). Springer. doi: 978-3540707127

Miguel, M. S., & Toral, R. (1997, July). Stochastic Effects in Physical Systems. Retrieved from `http://arxiv.org/abs/cond-mat/9707147v1`

Montbrió, E., & Pazó, D. (2011). Shear diversity prevents collective synchronization. *Phys. Rev. Lett.*, *106*, 254101.

Morelli, L. G., & Jülicher, F. (2007). Precision of Genetic Oscillators and Clocks. *Phys. Rev. Lett.*, *98*, 228101.

Pikovsky, A., Rosenblum, M., & Kurths, J. (2001). *Synchronization: A universal concept in nonlinear sciences*. Cambridge, UK: Cambridge University Press.

Quian Quiroga, R., Kraskov, A., Kreuz, T., & Grassberger, P. (2002). Performance of different synchronization measures in real data: A case study on electroencephalographic signals. *Phys. Rev. E*, *65*(4), 041903.

Torrence, C., & Compo, G. P. (1998). A practical guide to wavelet analysis. *Bull. Amer. Meteor. Soc.*, *79*(1), 61–78.

Verdugo, A., & Rand, R. (2008a). Center manifold analysis of a dde model of gene expression. *Commun. Nonlinear Sci. Numer. Simulat.*, *13*, 1112-1120.

Verdugo, A., & Rand, R. (2008b). Hopf bifurcation in a DDE model of gene expression. *Commun. Nonlinear Sci. Numer. Simulat.*, *13*, 235-242.

Wu, X. P., & Eshete, M. (2011). Bifurcation analysis for a model of gene expression with delays. *Commun. Nonlinear Sci. Numer. Simulat.*, *16*, 1073–1088.
